# Mercury and Arctic Char Gill Microbiota Correlation in Canadian Arctic Communities

**DOI:** 10.3390/microorganisms12122449

**Published:** 2024-11-28

**Authors:** Flora Amill, Patrice Couture, Nicolas Derome

**Affiliations:** 1 Institute of Integrative and Systems Biology, Laval University, Quebec, QC G1V 0A6, Canada; flora.amill.1@ulaval.ca; 2 Centre Eau Terre Environnement, Institut National de la Recherche Scientifique, 490 Rue de la Couronne, Quebec, QC G1K 9A9, Canada; patrice.couture@inrs.ca

**Keywords:** Arctic char, gill microbiota, 16s rRNA gene transcript, bacterial activity, mercury contamination, Canadian Arctic

## Abstract

Arctic char is a top predator in Arctic waters and is threatened by mercury pollution in the context of changing climate. Gill microbiota is directly exposed to environmental xenobiotics and play a central role in immunity and fitness. Surprisingly, there is a lack of literature studying the effect of mercury on gill microbiota. To fill this knowledge gap, our primary goal was to measure to what extent gill exposure to mercury may alter gill microbiota activity in Arctic char. Specifically, we calculated the correlation between the taxonomic distribution of gill-associated bacterial symbiont activity and total mercury concentration in livers and muscles in wild populations of Arctic char in the Canadian Arctic. Our results showed that total mercury concentrations in tissues were higher in Ekaluktutiak (Nunavut) than in the other sites in Nunavik. Proteobacteria was the main phylum correlated to mercury concentration in both tissues, followed by Bacteroidetes and Cyanobacteria. In the most contaminated sites, *Aeromonas* and *Pseudomonas* (Proteobacteria) were predominant, while mercury concentration negatively correlated with *Photobacterium* (Proteobacteria) or *Cerasicoccus* (Verrucomicrobia). In summary, we found that mercury contamination correlates with active gill microbiota composition, with potential implications of strains in modulating mercury toxicity, making them interesting for future biomarker studies.

## 1. Introduction

Arctic char, *Salvelinus alpinus* (Linnaeus 1758), iqaluk or tariungmiutaq in Inuktitut, is one of the main food sources and is culturally important for autochthonous populations in the North. It is also economically important all over Canada with fisheries and aquaculture [1,2,3]. It lives in cold and oligotrophic waters, could be landlocked or anadromous, and is widespread in the Canadian Arctic [4,5,6]. Unfortunately, the Arctic is facing multiple disturbances in the context of climate change and human activities. Among those numerous threats is the natural mercury cycle exacerbated by a warmer climate and human activities such as mining, electricity generation, or cement production [7]. Mercury is a contaminant released in the atmosphere through natural phenomena, such as volcanos or hydrothermal activities. However, 29 to 33% of the emissions are anthropogenic, and 56–65% come from terrestrial and aquatic sources [7,8]. Since the Industrial Revolution, this pollutant has increased in the atmosphere three to five times [9]. Once in the circulating atmosphere, mercury is distributed across the Arctic [10,11] and deposited in lakes, among other environments.

Arctic lake sediments are substantive mercury stocks. In sediments, methylating bacteria, including some sulfate-reducing or iron-reducing bacteria, can transform inorganic mercury into methylmercury when they carry a two-gene cluster *hgcA* and *hgcB* [12,13,14]. The methylmercury form is a neurotoxic contaminant that can bioaccumulate in the aquatic food web and biomagnify, threatening the food web and human health through fish consumption [10,15]. Autochthonous inhabitants of communities in the Arctic are particularly exposed to mercury contamination through food [16]. Monitoring surveys regularly assess the metal toxicity in fish tissues [17]. A 0.3 mg kg^−1^ concentration in the whole body was proposed as the limit above which the health and behavior of fish are negatively affected [18]. However, the limit for human consumption was set at 0.5 mg kg^−1^ [19]. Being a top predator in Arctic lakes, Arctic char is exposed to high levels of bioaccumulated methylmercury due to the biomagnification process [9], making it a good sentinel model in ecotoxicology. Top predator fish store total methylmercury in the liver and muscle tissues via blood [20,21]. Multiple evidence in other fish species shows that methylmercury is also bioaccumulated in other organs, including gills. The level of bioaccumulation could change among fish species and habitats. However, the liver usually has the highest mercury concentration, while the gills have the lowest. This is the case in cold saltwater Myctophid species living in the Southern Ocean [22] and in fish species in the Persian Gulf [23]. In another study on Nile tilapia (*Oreochromis niloticus*), which was exposed to different mercury concentrations in controlled conditions, the muscle was shown to be the organ containing the most mercury with a concentration of 5.72 mg kg^−1^ dry weight.

The gill showed negligible mercury accumulation (near zero) [24], but the damage caused by mercury exposure to the structure of the gill was reported. For example, mercury exposure caused gill injuries in Nile tilapia. They were pale, and histopathological damage, changes in lamella fusion, or even necrosis were noticed at high exposure concentrations [24]. Yellowfin seabream (*Acanthopagrus latus*), exposed to mercury, also showed physical effects on the normal structure of the gills, such as histopathological changes of the lamellar epithelium, the epithelial cells, and the filament epithelium, but also vascular alteration, or a decrease in the capability of gas exchange in fish [25]. Interestingly, alternation in gill histopathology could be a protective mechanism that prevents the entry of contaminants [26]. Overall, filtration through gills makes them particularly sensitive to chemicals from the surrounding environment [26,27].

Moreover, metal toxicity affects the microbial communities colonizing fish body surfaces (e.g., skin, gut, and gills), referred to as microbiota, through oxidative stress, DNA damage, and antimicrobial properties [27]. Mercury belongs to the list of stressors for teleost microbiota that can trigger dysbiosis, such as osmotic stress for anadromous fish [28,29], hypoxic stress [30,31], pathogen or parasitism stress [32,33,34,35,36,37], and other xenobiotic stress [38,39,40,41]. Dysbiosis occurs when external stress changes mucus protein composition, the immune system, or microorganisms’ dynamics, favoring opportunistic pathogen invasion [42,43]. However, environmental mercury could also induce selective change in teleost microbiota, promoting bacteria harboring genes related to a “*microbial metal resistome*”. The gill microbiota is thought to mitigate exposure to stressors [44], as documented in other tissue-associated microbiota [29,45,46]. Indeed, transcriptomic studies on marine Medaka (*Oryzias latipes*) and Amazonian fish gill microbiotas have shown that gene expression regulation differed during physical–chemical changes to overcome environmental stress [44,47]. Likewise, tolerant microorganisms may eliminate pollutants due to a panoply of genes enabling resistance and/or degradation of pollutants [12,48,49,50]. We focused on gill bacterial microbiota, as the gills constitute the first teleost semi-permeable barrier, exert a major role in water filtration, have a developed immune system, and are in direct contact with pathogens and exogenous contaminants, including mercury [51,52].

Wild anadromous Arctic char gut and skin microbiota have been characterized in the Arctic in the context of osmotic stress [28,53,54], and the first characterization of Arctic char gill microbiota has been published previously [55]. Here, we focused on the effect of mercury exposure of wild anadromous Arctic char on the condition status through the lens of active gill microbiota composition.

To reach this goal, we measured the correlation between total mercury in livers (*n* = 99) and muscles (*n* = 89) and the taxonomic distribution of gill microbiota activity using the 16S rRNA metabarcoding approach. Then, Spearman correlations were calculated between active bacterial genera in the Arctic char gill microbiota and the total mercury concentration. This analysis aimed to show which taxa in the gills could be sensitive or tolerant and possibly involved in mercury transformation. This study provides an unprecedented exploration of the correlation between mercury contamination and Arctic char gill microbiota in wild Arctic populations.

## 2. Materials and Methods

**Figure 1:**
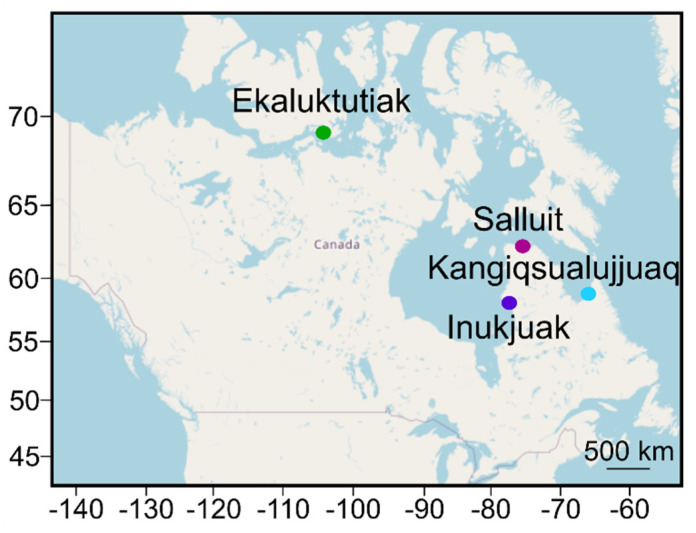
Maps of the four Inuit communities where Arctic char were collected. Ekaluktutiak, Salluit, Inukjuak, and Kangiqsualujjuaq were situated in four different hydrological basins in the Canadian Arctic: Cambridge Bay (Kitikmeot, Nunavut), Hudson Strait, Hudson Bay, and Ungava Bay (Kativik, Nunavik), respectively.

**Fish sampling.** Arctic char from four different regions were sampled across the Canadian Arctic (Ekaluktutiak (Cambridge Bay) in Nunavut and Salluit (Hudson Strait), Inukjuak (Hudson Bay), and Kangiqsualujjuaq (Ungava Bay) in Nunavik) (Figure 1).

Arctic char from Ekaluktutiak were collected with the Canadian High Arctic Research Station Campus (CHARS), Inuit guides from the Hunters and Trappers Organization (HTO), and Rautio Aquatic Laboratory (Université du Québec, Chicoutimi, QC, Canada) support. Fish from Salluit, Inukjuak, and Kangiqsualujjuaq have been caught thanks to the collaboration with the Northern Aquatic Resources laboratory at the Institute of Systems and Integrative Biology (IBIS, University Laval, Québec, QC, Canada) and the Ministry of Forests, Wildlife, and Parks (Québec, QC, Canada). Sampling campaigns occurred in the summer of 2018 and 2019, and all campaign details were reported earlier in Section 2 [55]. Gills were dissected and preserved in NAP buffer for microbiota analysis, and liver and muscle samples were collected for mercury analysis and preserved at −20 °C in tubes previously acid washed in 15% HNO_3_. We generated two tissue-specific datasets constrained by fish sample availability, resulting in 89 muscle (muscle dataset) and 99 liver (liver dataset) samples. In the liver dataset, Arctic char came from the four regions. In contrast, in the muscle dataset, only Arctic char from Ekaluktutiak, Salluit, and Kangiqsualujjuaq were caught to investigate the total mercury effect on gill microbiota. The two datasets were analyzed separately, and for each dataset, each sample underlies mercury data and gill microbiota data. In total, 51, 18, 25, and 5 liver samples were collected from Arctic char in Ekaluktutiak, Salluit, Inukjuak, and Kangiqsualujjuaq, respectively. A total of 63, 22, and 4 muscle samples were taken in Ekaluktutiak, Salluit, and Kangiqsualujjuaq, respectively. The sampled sites in Ekaluktutiak were located in the Greiner system: Greiner Lake; 69.18 N, −104.99 W, 36.9 km^2^ (n_liver_ = 20, n_muscle_ = 25), First lake; 69.20 N, −104.76 W, 3.16 km^2^ (n_liver_ = 6, n_muscle_ = 12), and Second lake; 69.18 N, −104.68 W, 268 km^2^ (n_liver_ = 11, n_muscle_ = 12), as well as the bay (n_liver_ = 7, n_muscle_ = 7) and the lake CBL5, named Inuhuktok; 69.25 N, −104.71 W, 1.11 km^2^ (n_liver_ = 7, n_muscle_ = 7) [56]. In Salluit, Duquet Lake (62.06 N, −74.53 W) was sampled with 18 liver and 22 muscle samples. In Inukjuak, only the livers were collected (n = 25) in Five Mile Inlet (58.56 N, −78.21 W), and finally, in Kangiqsualujjuaq, the river Koroc (58.89 N, −65.79 W) was harvested (n_liver_ = 5, n_muscle_ = 4). For those individuals, the Fulton index was calculated from morphological data (fork length and weight) with Froese’s equation: K=100∗(W/L)^3 (with weight (*W*) in g and length (*L*) in cm) [57].

**Total mercury (THg) analysis in liver and muscle samples.** Mercury analysis was carried out in Arctic char tissues (liver and muscle) at the Institut National de la Recherche Scientifique (INRS, Québec, QC, Canada). The samples were freeze dried (FTS Systems TMM, Kinetics Thermal Systems, Longueuil, QC, Canada; Freezone Plus 2.5 Liter Cascade Benchtop Freeze Dry Systems Labconco, Kansas City, MO, USA), and the total mercury in each sample was calculated with a direct mercury analyzer (DMA-80, Milestone Inc., Shelton, CT, USA) in triplicate weighing 0.10 g ± 0.02. A quadratic concentration was calculated, and a correction factor (0.80 ± 0.10) was calculated with the standards TORT-3 and DOLT-5 certified by the National Research Council of Canada (NRCC) to estimate the corrected quadratic total mercury concentration in the samples. Then, the triplicate mean was estimated, and an adjustment was made with the blanks to obtain the final total mercury dry weight concentration. Finally, the total mercury wet weight was calculated with a mean tissue-specific moisture content of 76% [58]. The Tort-3 and Dolt-5 recovery percentages were 99.4% and 99.68%, respectively. Samples from Five Mile Inlet (Inukjuak) were preserved in an NAP buffer. The THg concentration estimation was conducted by the “Centre Eau Terre Environment” laboratory at the INRS using the ICP- MS methods with Indium as the internal standard. Finally, some samples from Ekaluktutiak in 2018 were analyzed by the Laboratory of Environment and Climate Change Canada at the National Laboratory for Environmental Testing (Burlington, ON, Canada).

**rRNA 16s analysis.** To focus on the taxonomic distribution of microbiota activity instead of the taxonomic distribution of bacterial abundance, a 16S rRNA gene metabarcoding approach was performed on total RNA instead of DNA. Sequence reads were filtered and trimmed using dada2 [59] with R v.3.2.2 [60]. Filtration options were the same as in [55]. Briefly, truncations were made using Phred scores (at 275 for forward reads and 270 for reverse reads), Nas and chimeras were removed and the same threshold of expected error for the forward reads (4) and the reverse reads (5) and the same prediction model were used, and ASVs were decontaminated with control reads. The identity threshold of 97% was applied to cluster ASVs with dada2. From the ASV raw counts, metadata, and taxonomic tables, two phyloseq objects for liver (n = 99) and muscle (n = 89) data were constructed using the package phyloseq [61]. ASVs with low abundance (<1 × 10^−5^) and three samples with very low total count (<10,000) were removed from both phyloseq objects. In the liver phyloseq object, one of the samples came from Cambridge Bay in Ekaluktutiak, one from Duquet Lake in Salluit, and one from Five Mile Inlet in Inukjuak. For the muscle phyloseq object, the removed samples were the same sample from Ekaluktutiak and two samples from Salluit (including the sample removed in the liver object). After filtrations and normalization, 71,191 and 70,347 reads were retained in the liver (n = 96) and muscle phyloseq (n = 86) objects, respectively.

### Statistics

**Mercury and Fulton Index correlation.** Mercury concentrations in the liver and dorsal muscle and Fulton index boxplots were built using the function “ggboxplot” from the package “ggpubr” [62]. To assess the relationship between the mercury concentration (in livers and muscles) and the Fulton index, Spearman correlations were performed with the “cor.test” function and the method “spearman” in Rstudio (v 4.0.5). Differences between geographical groups of the Fulton index and the mercury concentrations in the liver and muscle were statistically estimated with the Kruskal–Wallis test and multiple pairwise comparisons between groups using the functions “Kruskal.test” and “pairwise.wilcox.test”, respectively.

**Relative transcriptomic activity**. Means of relative transcriptomic activity of the 100 most active taxa in Arctic char gills at the genus level were visualized in a barplot constructed with the package ggplot2 in R [63]. The Kruskal–Wallis test, followed by the Wilcoxon test corrected with Benjamini–Hochberg correction, were used to assess significant differences in the relative abundance of the six most abundant genera between regions in both datasets.

**Beta diversity.** To assess the differential bacterial composition in the Arctic char gills between the geographical sites, ordination relied on UniFrac weighted distances made for the two datasets. Non-metric Multidimensional Scaling (NMDS) plots with mercury parameters fitted were performed using the packages dplyr, ggplot2 [64], and the envfit function in the vegan package in Rstudio [65]. The latter made multiple regressions with 9999 permutations, and the *p*-value was adjusted using the Bonferroni correction with the function p.adjust. To statistically understand the different bacterial compositions between groups, a permutation-based multivariate analysis of variances (PERMANOVA) was performed using the adonis function in the vegan package in Rstudio [65]. An adjustment with the Benjamini–Hochberg correction has been made to avoid misinterpreting the results because of the unbalanced experimental design [66]. Finally, an analysis of multivariate homogeneity of group dispersion (variances) was performed with the betadisper function in the vegan package and a boxplot of the distances to the centroid.

**Spearman correlations between bacterial genus and mercury concentrations.** Associations between mercury concentrations (in muscles and livers) and bacteria activity at a genus rank were made using Spearman correlations with the “rcorr” function in the package “Hmisc” [67] in Rstudio (v 4.0.5). Correlations with a coefficient > |0.4| and a *p*-value adjusted with Bonferroni < 0.05 were kept and visualized in networks built on Cytoscape (v 3.5.1) [68]. Genera were represented by nodes, with their size depending on their activity and color depending on their phylum. Each significant correlation between a genus and mercury concentration in the muscle or the liver was represented by red or green edges for negative or positive correlations, respectively. Edges size reflects the importance of the correlation. The thicker the line is, the greater the correlation coefficient. Finally, the same network with the Spearman correlations filtered to be higher than |0.5| was made for the liver dataset but not for the muscle dataset, where no correlation with a coefficient higher than |0.5| was found. All the figures were aesthetically adjusted using Inkscape [69].

## 3. Results

**Figure 2:**
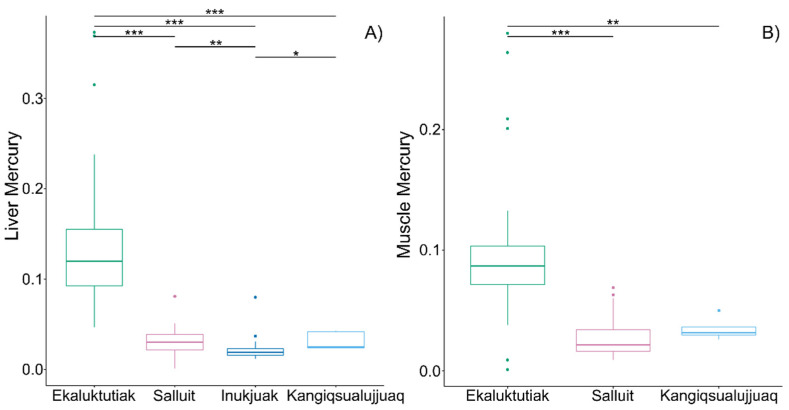
Boxplot of mercury concentrations (mg kg^−1^) wet weight in Arctic char tissues. Mercury content in the liver (**A**) was measured in Ekaluktutiak, Salluit, Inukjuak, and Kangiqsualujjuaq, while mercury content in the dorsal muscle (**B**) was assessed only in Ekaluktutiak, Salluit, and Kangiqsualujjuaq. Statistical significance: “***” *p* < 0.001, “**” *p* < 0.01, “*” *p* < 0.05.

**Different Fulton index and mercury content according to the different geographical sites.** Mean comparisons between groups for the Fulton index using the Kruskal–Wallis test (ꭕ^2^ = 17.90, *p* < 0.001) followed by multiple pairwise comparisons have shown significant differences between Ekaluktutiak and Inukjuak (*p* = 0.001) (Table 1, Figure S2). Moreover, the Kruskal–Wallis test also showed a significant difference in mercury concentration in the liver among the four sites (ꭕ^2^ = 73.59, *p* < 0.001). Multiple pairwise comparisons between groups revealed that Ekaluktutiak had significantly higher mercury concentration in the liver than Salluit, Inukjuak, and Kangiqsualujjuaq (*p* < 0.001). Then, in Nunavik, Inukjuak was significantly lower than Salluit (*p* = 0.01) and Kangiqsualujjuaq (*p* = 0.02) (Figure 2A, Table 1). There were also significant differences between groups in mercury concentration in the muscle (Kruskal–Wallis: ꭕ^2^ = 43.81, *p* < 0.001). More precisely, samples from Ekaluktutiak had significantly higher mercury concentrations than samples from Salluit (*p* < 0.001) and Kangiqsualujjuaq (*p* = 0.004) (Figure 2B, Table 1).

Moreover, Ekaluktutiak was the only site where four samples exceeded the liver’s concentration limit (0.3 mg kg^−1^), which is deemed to induce a toxic effect on fish [18]. Three samples came from the Second lake and one from Cambridge Bay (Figure S1). However, none of the muscle and liver samples exceeded the threshold for human consumption of 0.5 mg kg^−1^ [18]. Finally, negative Spearman correlations were detected between the Fulton index and mercury concentration in the liver (rcorr = −0.45, *p* < 0.001) and muscle (rcorr = −0.27, *p* = 0.01).

**Figure 3:**
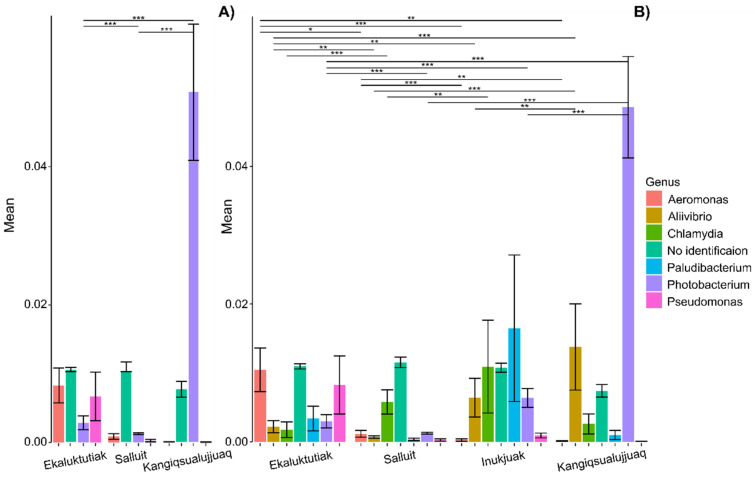
Relative abundance of the 100 most active ASVs at a genus rank in the gill microbiota of the Arctic char in the muscle dataset (n = 96, 4 communities) (**A**) and the liver dataset (n = 86, 3 communities) (**B**). Statistical significance: “***” *p* < 0.001, “**” *p* < 0.01, “*” *p* < 0.05.

**Different relative activity of the most active bacterial genera in the Arctic char gill microbiota.** When focusing on the 100 most active ASVs at the genus rank, three genera were represented in the muscle dataset (*Aeromonas*, *Photobacterium*, and *Pseudomonas*) (Figure 3A), and six genera were represented in the liver dataset (*Aeromonas*, *Aliivibrio*, *Chlamydia*, *Paludibacterium*, *Photobacterium*, and *Pseudomonas*) (Figure 3B).

*Aeromonas* activity was significantly different between geographical sites in the liver dataset (Kruskal–Wallis: ꭕ^2^ = 43.81, *p* < 0.001) and was being significantly higher in Ekaluktutiak than in Salluit (*p* = 0.02), Inukjuak (*p* < 0.001), and Kangiqsualujjuaq (*p* = 0.002) (Figure 3B). In the muscle dataset, the mean relative activity of *Aeromonas* was marginally higher in Ekaluktutiak than in the other groups (Figure 3A) (Kruskal–Wallis: ꭕ^2^ = 5.68, *p* = 0.06). *Photobacterium* activity differed significantly between groups in both the liver (Kruskal–Wallis: ꭕ^2^ = 209.99, *p* < 0.001) and muscle (Kruskal–Wallis: ꭕ^2^ = 180.38, *p* < 0.001) datasets. *Photobacterium* was significantly more abundant in Kangiqsualujjuaq than in Ekaluktutiak, Salluit, and Inukjuak for both datasets (*p* < 0.001). *Aliivibrio* activity was significantly higher in Kangiqsualujjuaq than in Ekaluktutiak (*p* < 0.001), Salluit (*p* = 0.01), and Inukjuak (*p* = 0.01). Finally, in the liver dataset, *Paludibacterium* and *Chlamydiae* had higher activity in Inukjuak. However, only *Chlamydiae* showed significant differences between groups (Kruskal–Wallis test: ꭕ^2^ = 21.84, *p* < 0.001), with significantly higher activity in Salluit than in Ekaluktutiak (*p* < 0.001) and significantly higher activity in Inukjuak than in Salluit (*p* = 0.002).

**Figure 4:**
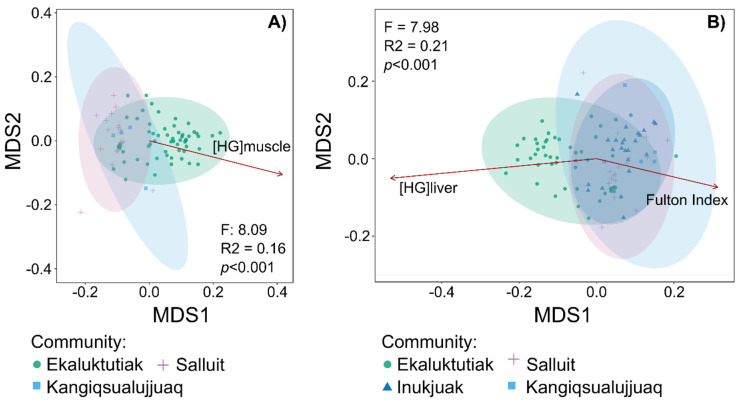
NMDS plots relied on UniFrac weighted distances calculated in two different datasets: the muscle dataset with the communities Ekaluktutiak (green circle), Salluit (pink cross), and Kangiqsualujjuaq (turquoise blue square) (**A**) and the liver dataset with samples from the three same communities and Inukjuak (dark blue triangle) (**B**). Mercury concentrations in the liver and muscle and the Fulton index were fitted on the NMDS plots in red arrows.

**The different taxonomic distribution of bacterial activity in Arctic char gill microbiota is correlated with bioaccumulated mercury.** NMDS plots with mercury parameters fitted (Figure 4) were used to assess the correlation between mercury content in both the liver and muscle and the differential composition of the active gill microbiota between sampling sites.

Ekaluktutiak seemed to have a different bacterial composition from Salluit and Kangiqsualujjuaq for both organs (Figure 4). In the liver dataset, Ekaluktutiak’s bacterial composition also differed from Inukjuak’s (Figure 4B). The PERMANOVA showed a significant composition difference between groups in the muscle dataset (F = 8.09, R^2^ = 0.16, *p* < 0.001), as well as in the liver dataset (F = 7.98, R^2^ = 0.21, *p* < 0.001). In both datasets, Ekaluktutiak, in green (Figure 4), was separated from Salluit and Kangiqsualujjuaq (multiple pairwise comparisons: *p* < 0.01). Furthermore, Ekaluktutiak significantly differed from Inukjuak in the liver dataset (multiple pairwise comparisons: *p* < 0.01). This difference was correlated to the mercury concentration in both the muscle and liver (R^2^ = 0.17, *p* < 0.001, and R^2^ = 0.29, *p* < 0.001, respectively). Therefore, it suggests that the mercury content could have influenced the taxonomic distribution of Arctic char gill bacterial symbiont activity. Finally, the parameter “Fulton index” was significantly correlated with the bacterial composition (R^2^ = 10, *p* = 0.008) in the liver dataset. This was not the case for the muscle dataset.

**Figure 5:**
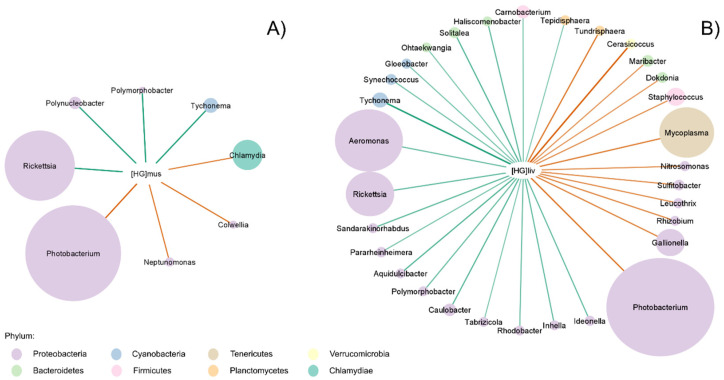
Spearman correlations between bacterial genera abundance and mercury concentrations in the muscle (**A**) and liver (**B**) are represented in a network with a minimum coefficient of **|**0.4**|** and a *p*-value adjusted with Bonferroni of 0.05. Each node represents one genus; its size depends on the abundance, and its color changes according to its phylum. Green edges represent positive correlations, while red edges represent negative correlations. Thicker edges represent stronger Spearman correlations.

**Bacterial genera positively and negatively correlated to mercury concentration.** Figure 5 graphically represents significant Spearman correlations higher than |0.4| between bacterial genera activity and mercury concentration.

Proteobacteria was the main phylum that had a significant relationship with mercury concentration. More precisely, a strong negative correlation between *Photobacterium* and mercury content was detected in both liver and muscle networks (Figure 5A,B and Figure S3), with rcorr = −0.50 and *p <* 0.001. Another genus belonging to Cyanobacteria, *Tychonema*, was significantly positively correlated to the mercury concentration (*p* < 0.001) in all networks (Figure 5A,B and Figure S3) with rcorr = 0.53, 0.67, and 0.68, respectively. Likewise, strong correlations (*p >* 0.001) between *Rickettsia* and mercury concentrations in both liver and muscle were found with rcorr = 0.42 and 0.48, respectively (Figure 5A,B). *Chlamydia* (Chlamydiae) was significantly negatively correlated to muscle mercury concentration (rcorr = −0.41, *p* < 0.001) (Figure 5A). Liver mercury concentration was positively correlated to the Proteobacteria *Aeromonas* (rcorr = 0.47, *p* < 0.001), *Aquidulcibacter* (rcorr = 0.52, *p* < 0.001), and *Caulobacter* (rcorr = 0.51, *p* < 0.001) but also to the Bacteroidetes *Solitalea* (rcorr = 0.52, *p* < 0.001). Finally, *Mycoplasma* (Tenericutes) (rcorr = −0.49, *p* < 0.001) was negatively correlated to the liver mercury concentration (Figure 5B).

## 4. Discussion

The present study assessed the correlation between mercury concentration (in the muscle and the liver) and the active gill bacterial microbiota of wild Arctic char populations. Fish samples were collected in Ekaluktutiak (Victoria Island, Nunavut), Salluit (Hudson Strait, Nunavik), Inukjuak (Hudson Bay, Nunavik), and Kangiqsualujjuaq (Ungava Bay, Nunavik). Fish from Ekaluktutiak, the northernmost Inuit community, showed the highest mercury concentrations in both hepatic and muscular tissues. Our data suggest that mercury concentration is significantly correlated with the taxonomic distribution of active gill bacterial symbionts. Among these bacterial symbionts, activity levels of four and nineteen genera correlated positively (|0.4|) to mercury concentration in the muscle and liver.

### 4.1. Higher Mercury Concentrations at the Highest Latitude

In Nunavut, in the community of Ekaluktutiak, Arctic char were collected from four lakes and Cambridge Bay, while in Nunavik, samples came from one lake in Salluit and three rivers in Inukjuak and Kangiqsualujjuaq (Figure S1). Mercury concentrations in Arctic char’s tissues varied across locations and were significantly higher in Nunavut sites (Figure 2). Moreover, four fish livers from Ekaluktutiak (Nunavut) exceeded 0.3 mg kg^−1^ wet weight of mercury concentration, a toxicity threshold for fish health determined by Dillon et al., 2010. However, they did not reach the toxicity threshold of 0.5 mg kg^−1^ wet weight from Health Canada’s guideline for human consumption of fish [18,19]. Interestingly, we detected a negative correlation between the Fulton index of Arctic char and mercury concentrations in the muscles and livers in the Nunavut and Nunavik sites. The negative relationship between metal-contaminated fish and the Fulton index was previously documented in yellow perch and anadromous Arctic char [70,71,72]. A negative relationship was also measured in anadromous populations of Arctic char from Duquet and Françoys-Malherbe lakes (Salluit, Deception Bay, Nunavik) for total mercury in Arctic char muscles (Pearson correlation coefficient = −0.04) but was not significant (*p* = 0.66) [73]. Beyond the concentration thresholds mentioned above [18,19,74], methylmercury was documented to induce acute toxic effects in Arctic char (with liver necrosis [75] or hepatic fibrosis [76]) and yellow perch (with growth, reproduction, or developmental disorders [77]). We can hypothesize that this negative correlation found in our data showed that higher mercury concentrations in the tissues could reduce the somatic conditions of Arctic char. In the review by Chételat (2015), they also supported the hypothesis that weak somatic conditions (and slower growth rate) positively correlate to mercury bioaccumulation in landlocked char in Kitikmeot [78].

Yet, the literature does not support the potential correlation between higher latitudes and higher concentrations of bioaccumulated mercury in fish. On the one hand, landlocked Arctic char were reported to have higher concentrations of total mercury in lower latitudes [79], potentially due to higher atmospheric mercury deposition in lower latitudes [80]. On the other hand, another study did not show any correlation between total mercury concentration and latitude [81]. In our case, other factors that co-occur with our latitudinal gradient should be studied to determine further the cause of higher mercury contamination in fish from Ekaluktutiak compared to the more southern Nunavik fish populations.

Several parameters in the Canadian Arctic associated with mercury concentration in Arctic char can be linked to the differences at large spatial scales. First, morphological traits such as size and age, as well as different life cycles (landlocked or anadromous), diets, or trophic positions (top predators) [76,79,81,82], can influence mercury content in fish, including Arctic char. Secondly, the immediate environment, including wetland coverage [83], watershed size [81], and water chemistry, including temperature [84,85], can all interfere with mercury deposition and transformation in lakes. Climate parameters, including precipitation, seasonal ice loss, permafrost thaw, and landscape diversity [7,73,85,86], could also affect the dynamics and structure of the lacustrine ecosystem, therefore impacting mercury inputs in lakes and rivers. Finally, local and mid-latitude human activities have an impact on mercury emissions and terrestrial deposition, such as mining, cement production, silver or gold extraction, burning of fossil fuels, or waste incineration [7,85,87]. Challenging waste treatment [88] could also bring high loads of nutrients into lakes, which could fuel anoxic methylating bacteria, such as the anaerobic sulfate-reducing bacteria (SRB) and iron-reducing bacteria (FeRB), which contain the methylation genes hgcAB [14,89]. This could lead to methylmercury formations in sediments [90,91,92] or water columns [93] in anoxic conditions [94].

We did not have enough data to measure the different environmental influences explaining why the mercury concentration in Arctic char’s tissues was higher in Ekaluktutiak than in Salluit, Kangiqsualujjuaq, and Inukjuak. Moreover, the studied lakes in Ekaluktutiak were oligotrophic [95]. We hypothesize that temperature, which was higher in Ekaluktutiak than in the other groups [56], may have played an important role in facilitating the bioaccumulation of mercury in these Arctic char, as there is a positive relationship between temperature and mercury methylation rates, which results from enhanced microbial activity [88].

Mercury concentrations in Ekaluktutiak were still low compared to those in Great Lakes region fishes. For instance, yellow perch (*Perca flavescens*) had 0.01–2.60 µg g^−1^ Hg of wet weight in the muscle [77] compared to 0.001–0.370 µg g^−1^ Hg of wet weight in livers and 0.001–0.280 µg g^−1^ Hg of wet weight in muscles in our study. Furthermore, we must remember that the concentration of total mercury measured in summer (our season of sampling) decreases in winter [73] and that anadromous Arctic char present less mercury contamination than landlocked Arctic char [96]. Finally, total mercury concentration in anadromous Arctic char significantly decreased between 2004 and 2013, possibly due to the bio-dilution of the metal in the context of warmer springs [90,91,92,96]. Still, mercury exposure was inversely correlated to fish body condition, thus suggesting that direct or indirect adverse effects on fish health are ongoing. On the other hand, in Nunavik, even if mercury concentrations were lower in fish compared to Nunavut, Kangiqsualujjuaq, Inukjuak, and Salluit lakes and rivers are still deemed at risk for mercury contamination because, for example, of mining. Monitoring environmental contamination, including rivers and fish, as organized by scientific and local communities, should continue to detect early signals of mercury exposure in aquatic ecosystems [73,93,94,95].

### 4.2. Correlation Between Mercury Concentrations and Arctic Char Gill Microbiota

Mercury is a toxic contaminant and affects fish in different ways. Studies on fathead minnows (*Pimephales promelas*) in North American freshwater ecosystems have shown developmental consequences on exposed eggs with neurological effects [97,98]. Mercury toxicity (on development, reproduction, behavior, or brain injury) has also been suspected at early life stages in sheepshead minnow (*Cyprinodon variegatus*) [99], zebrafish (*Danio rerio*), yellow perch [100,101], or common carp (*Cyprinus carpio*) [102]. Cheaib et al., 2020 also saw the impacts of the toxic metal cadmium on the bacterial community assembly and structure of the yellow perch skin and gut microbiota, leading to dysbiosis [40]. In gills, the metal impact on microbiota is poorly documented in fish, and no research has investigated the effect of mercury on gill microbiota. However, Zhou et al. (2023) described the impact of oxidative stress induced by copper sulfate, a component usually used in aquaculture, on gill microbiota in yellow catfish (*Pelteobagrus fulvidraco*) [103]. The results showed that the composition and diversity of the gill microbiota were disturbed, leading to dysbiosis. They also reported decreased antioxidant enzyme activity inducing oxidative stress in the gill and immunosuppression disturbing the yellow catfish immune system. Overall, pathogens seemed more abundant in the microbiota of organisms living in contaminated environments [40,50,104]. Here, we measured mercury’s association with the bacterial activity in Arctic char gill microbiota. We noticed that the most contaminated Arctic char population coming from Ekaluktutiak also showed predominance in the activity of the genera *Aeromonas* and *Pseudomonas* in gill microbiota (Figure 2 and Figure 3). Interestingly, those genera contain many opportunistic pathogens. Under stressed conditions, *Aeromonas* sp. could trigger septicemia and furunculosis [105,106], and *Pseudomonas* could trigger infections [31,42]. *Aeromonas* abundance was already found to increase in the common carp gut microbiota after mercury exposure and to induce potential brain injury [102]. *Pseudomonas* sp. increase had also been reported in common carp gut microbiota with copper exposure [104], and *Pseudomonas brenneri* was isolated from a mining effluent containing heavy metal ions [107]. Knowing that those two genera contain numerous fish opportunistic pathogens [31,42,108,109,110,111] and that mercury moved bacterial composition towards these opportunistic genera, it may suggest that mercury bioaccumulation contributes to the rise of potential pathogens in Arctic char gill microbiota.

Mercury induces selective pressure on microorganisms’ communities. On the one hand, metals contain antimicrobial properties and could kill vulnerable strains [27]. On the other hand, tolerant microorganisms can adapt and present a “microbial metal resistome”, a term used to characterize all metal resistance genes in one environment [112]. Thus, for those tolerant species containing metal resistance genes, contamination could be a benefit for them to reach more resources, as already shown for pesticide-resistant genera [113]. However, the capacity of a resilient species to adapt, recover from exogenous disturbances, and maintain a stable state will depend on the amount and frequency of the metal input [40,114,115]. However, if contaminants modulate the microbiome, the reciprocal is also true, and the microbiome also has a role in toxicity modulation [116].

### 4.3. Mercury Transformation Role of Microbiota

Mainly, microorganisms transform inorganic mercury into bioaccumulative methylmercury through methylation. Notably, some sulfate-reducing and Fe(III)-reducing bacteria play a role in mercury bioavailability [13,48,117]. Indeed, a large panel of anaerobic bacteria, including the phylum Deltaproteobacteria or Firmicutes, contain genes involved in methylation, such as *hgcAB* [14,48,116,117,118]. Bacterial methylmercury productions are likely to occur in thawing permafrost soils, saturated agricultural soils, or anaerobic environments [48] and are reported in gut microbiota in teleost [119]. In human gut microbiota, 17 bacterial genera have been reported as methylmercury biomarkers [120], but none were found in gill fish microbiota datasets. Yet, most of the bacterial genera active in Arctic char gill microbiota, correlated to mercury concentration in livers and muscles, belonged to the phylum Proteobacteria (Figure 5). Hao et al., 2021 mention that Proteobacteria contain heavy metal resistance genes and represent a resistant phylum to metal-polluted environments [112]. Actinobacteria, Firmicutes, and Bacteroidetes (among others) also contain resistance genes [112] and are found in abundance in Arctic char gill microbiota [55]. More precisely, we found that *Tychonema*, *Solitalea*, *Aquidulcibacter*, and *Caulobacter* had the strongest positive relationships with mercury concentration in livers. *Tychonema* is a Cyanobacteria that produces a cyanotoxin [121] and has been reported in macroinvertebrate microbiota showing dysbiosis and living in wastewater effluent [122]. This genus was also abundant in a controlled experiment with a metal mixture environment and high temperatures [123]. *Solitalea* is a Bacteroidetes obligately aerobic or facultative anaerobic [124] and a nitrate-reducing bacteria [125] previously reported in a polluted pond near an abandoned coal mine drainage site [126]. *Aquidulcibacter* is a Proteobacteria that can live in eutrophic lakes [127]. Finally, the Proteobacteria *Caulobacter* was described in the literature as a genus that can harbor metal resistance genes against oxidative stress [128,129]. Mercury concentrations in muscles were also the most positively correlated to the genus *Polymorphobacter* and *Rickettsia*, both Proteobacteria. *Polymorphobacter* was discovered in the Antarctic [130] and found in contaminated sites near abandoned mines [131]. Finally, a link between heavy metal pollution and *Rickettsia* sp. infection in the digestive and respiratory organs of sea cucumbers was made by Elghazaly and Ghoneim, 2017 [132]. All those genera seem to harbor tolerant strains that evolve in metal-contaminated sites. Further transcriptomic investigation on metal gene resistance of those genera could be interesting to understand how a bacterial species can be tolerant to metal or, in our case, trace metal, and if it has a role in mercury methylation.

Also, one biotic process that degrades methylmercury is demethylation, which is undertaken by bacterial genes. Some aerobic or facultative anaerobic bacteria were reported to contain a Hg resistance mer-operon involved in a demethylation pathway producing Hg(0) and CH_4_ [133,134]. The gene merA allows mercuric reduction, merB is involved in organomercurial cleavage, and merP, merT, and merC genes are mercuric ion transports [123] and references cited. For example, genomes from *Pseudomonas* species and *Staphylococcus* strains isolated from contaminated soil harbored a merB variant [133]. Interestingly, *Pseudomonas* activity was significantly higher in Ekaluktutiak, where fish had the highest mercury concentration in both the muscle and liver (Figure 3). However, no correlation between mercury concentration and this genus was detected. Moreover, our data found that *Staphylococcus* activity was negatively correlated with mercury concentrations (Figure 5). Knowing that *Staphylococcus* was one of the 50 most active genera in Arctic char gill microbiota in Inukjuak [55] and that some strains could harbor a demethylation gene, we are wondering if a demethylation process could be attributed to *Staphylococcus* and explain the lowest mercury concentrations in the livers in Arctic char coming from Inukjuak (Figure 2A). However, because total mercury was not measured in the different environments of this study, we cannot state whether the lowest mercury concentration in fish tissues resulted from low mercury concentration in the environment or if bacterial processes in gills could have demethylated this contaminant. Still, it is interesting to know that Hudson Bay has been reported to be a sampling site harboring the lowest total mercury in water column and sediments in the Canadian Arctic (after the Arctic Archipelago) and that methylmercury concentration was low in snowpack [135] and references cited. Mercury concentrations in the liver were the most negatively correlated to *Cerasicoccus* from the phylum Verrucomicrobia and the Proteobacteria *Photobacterium*. Moreover, *Photobacterium* was also the most negatively correlated genus to mercury concentration in the muscle. *Photobacterium* is a common genus in salmonid microbiota [136,137,138], and *Photobacterium phosphoreum* has already been used as a sentinel microorganism for heavy metal contamination [139]. Although *Cerasicoccus* is poorly described in the literature, there is evidence that metagenome-assembled genomes (MAGs) of Verrucomicrobiae sampled in the Arctic Ocean are potentially involved in mercury reduction and have the merA gene [140]. Apart from the mer operon system, some bacteria can reduce mercury bioaccumulation by binding the metal (chelation), which neutralizes the stressor in the fish gut [112] and references cited.

Unfortunately, due to challenging sampling conditions in the Arctic, collecting the same environmental parameters in all sites was not possible for this study. Therefore, we prioritized the highest geographical coverage by focusing on mercury concentration in fish. Many other parameters may have a vast synergic impact on the chemical process, leading to mercury bioaccumulation and transformation, as evidenced in other fish habitats, such as microorganisms recruited from the diet. Indeed, according to the diet in controlled experiments, either methylation or demethylation was induced in three fish host gut microbiota: yellowstripe gobyfish (*Mugilogobius chulae*), largemouth bass (*Micropterus salmoides*), or yellowfin seabream [119,141,142]. The Arctic char gut microbiota would play an important role in the biotransformation of mercury. For instance, allochthonous bacterial strains recruited from the diet could mitigate mercury toxicity: zooplankton, an important food source for Arctic char, have been reported to harbor the hgcA gene in the Baltic Sea [143]. Gill microbiota would be expected to play a major function in mitigating Hg toxicity in fish, as gills are in direct contact with the environment, filter contaminants, and, in turn, are exposed to allochthonous microorganisms potentially harboring mercury resistance genes [140]. Active recruitment of allochthonous microorganisms in gills for adaptation to a stressing environment was demonstrated in the zebrafish model [44] and observed in natural populations of four species of Amazonian fish [144]. Therefore, more investigation of the gill microbiota’s role in biotransformation is needed to assess the gill microbiota in detoxifying methylmercury.

## 5. Conclusions

This paper highlights mercury pollution’s importance in Arctic aquatic ecosystems and their two-way relationship with Arctic char gill microbiota. Pollutants could disrupt the teleost microbiome, inducing dysbiosis and altering fitness. In return, the microbiome could interfere with the toxicity of the pollutant. Only gut microbiota has been investigated for metal contamination, but this topic remains scarce. Here, we show that the Arctic char gill microbiota is also correlated with mercury contamination and contains some tolerant bacteria capable of modulating mercury toxicity. More investigations on teleost microbiota and the role of bacteria in mercury biotransformation are important for a few reasons. First, bacterial methylation rate measurement would aid in predicting potential methylmercury concentrations in teleost [145]. Then, probiotics could be developed to avoid metal or metalloid contamination in teleost or human tissues [135]. Already, gut remediation has been proposed to develop probiotics for contaminant bioremediation [50]. Thus, teleost gill microbiota could also harbor interesting bacteria for bioremediation in contaminated sites.

## Figures and Tables

**Table 1:** Fulton index and mercury concentrations in the liver and muscle of Arctic char at Ekaluktutiak, Salluit, Inukjuak, and Kangiqsualujjuaq. Means with standard deviation; the minimum and maximum values are reported for the Fulton index and mercury concentration (mg kg^−1^) in liver and muscle samples. The non-parametric test Kruskal–Wallis followed by the multiple pairwise comparisons between groups with the *p*-value adjusted with Benjamini–Hochberg was performed for both parameters. Letters a, b, c, d, e, and f represent the significance between groups.

Communities	Region	Nliver	Nmuscle	Stat	Fulton Index	[Hg] Liver	[Hg] Muscle
**Ekaluktutiak**	Victoria Island, Kitikmeot (Nunavut)	51	63	**Mean ± SD**	1.0 ± 0.3 ^a^	0.14 ± 0.08 ^bc^	0.09 ± 0.05 ^ef^
				**Min**	0.2	0.05	0.001
				**Max**	1.6	0.37	0.28
**Salluit**	Hudson Strait, Kativik (Nunavik)	18	22	**Mean ± SD**	1.1 ± 0.1	0.03 ± 0.02 ^b^	0.03 ± 0.02 ^e^
				**Min**	0.8	0.001	0.01
				**Max**	1.4	0.08	0.07
**Inukjuak**	Hudson Bay South, Kativik (Nunavik)	25	NA	**Mean ± SD**	1.2 ± 0.1 ^a^	0.02 ± 0.01 ^bd^	NA
				**Min**	0.9	0.01	NA
				**Max**	1.3	0.08	NA
**Kangiqsualujjuaq**	Ungava Bay, Kativik (Nunavik)	5	4	**Mean ± SD**	1.2 ± 0.2	0.03 ± 0.01 ^cd^	0.03 ± 0.01 ^f^
				**Min**	0.9	0.02	0.03
				**Max**	1.3	0.04	0.05

## Data Availability

Raw sequence reads from this study were deposited in the NCBI Sequence Read Archive under the following BioProject number: PRJNA944667.

## Supplementary Materials

The following supporting information can be downloaded at https://www.mdpi.com/article/10.3390/microorganisms12122449/s1, Figure S1: Detailed maps of the fishing sites in the four Inuit communities: Ekaluktutiak (A), Salluit (B), Inukjuak (C), and Kangiqsualujjuaq (D). Biooracle on Rstudio and Inkscape were used to create this map. Figure S2: Boxplot of the Fulton index in the four Arctic char communities: Ekaluktutiak, Salluit, Inukjuak, and Kangiqsualujjuaq. Statistical significance: ‘****’ p* < 0.001. Figure S3: Network of Spearman correlations between bacterial genus abundance and mercury concentrations in the liver with a minimum coefficient of |0.5| and a p-value adjusted with Bonferroni of 0.05. Genera are represented by nodes, and their size and color change according to their abundance and phylum, respectively. Green edges represent positive correlations, while red edges represent positive correlations, and the thicker the edge is, the stronger the Spearman correlation is.
