# Supplementary material for: Mercury and Arctic Char Gill Microbiota Correlation in Canadian Arctic Communities
Source: Microorganisms. 2024 Nov 28;12(12):2449. doi: 10.3390/microorganisms12122449 (PMC11678572; doi:10.3390/microorganisms12122449)
Supplement: Supplementary file 1 [file microorganisms-12-02449-s001.zip › microorganisms-3241192-supplementary.pdf]

Mercury and Arctic char gill microbiota correlation in Canadian Arctic communities

Flora Amill [a], Patrice Couture [b], Nicolas Derome [a]

a. Institute of Integrative and Systems Biology, Laval University, Quebec, Canada
b. Institut National de la Recherche Scientifique – Centre Eau Terre Environnement, 490 Rue de la Couronne Québec (Québec), Canada, G1K 9A9

Corresponding author: flora.amill.1@ulaval.ca

Supplementary materials

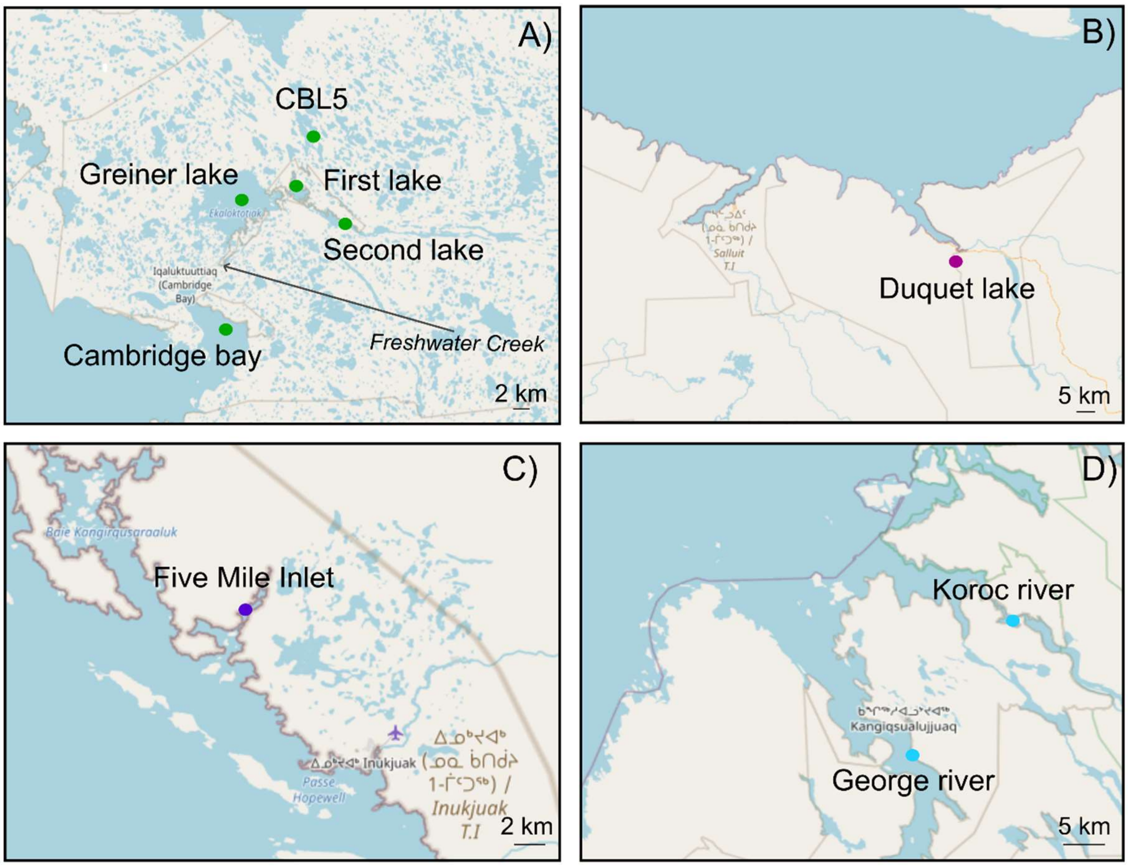

Figure S1*: Detailed maps of the fishing sites in the four Inuit communities: Ekaluktutiak (A), Salluit (B), Inukjuak (C), and Kangiqsualujjuaq (D). Biooracle on Rstudio and Inkscape were used to create this map.*

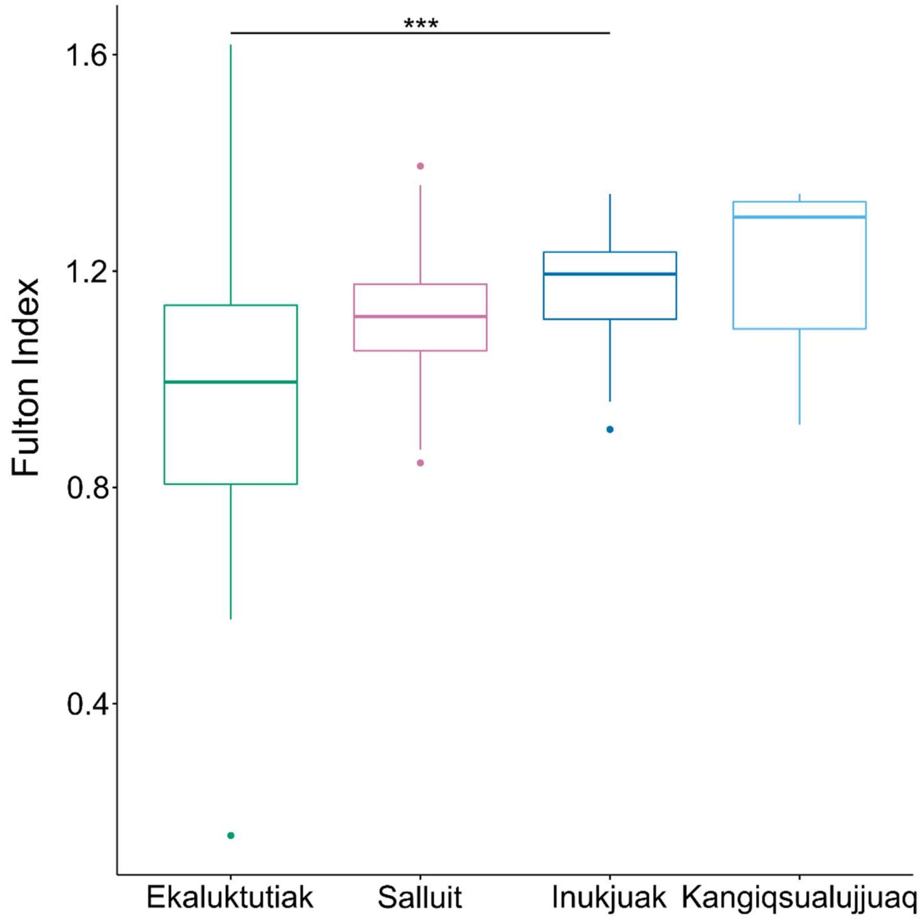

Figure S2: Boxplot of the Fulton index in the four Arctic char communities: Ekaluktutiak, Salluit, Inukjuak, and Kangiqsualujjuaq. Statistical significance: '***' $p < 0.001$.

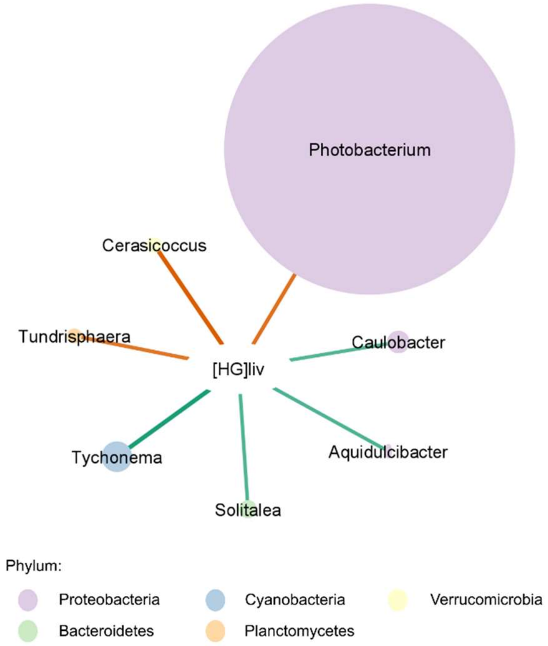

Figure S3. Network of Spearman correlations between bacterial genus abundance and mercury concentrations in the liver with a minimum coefficient of |0.5| and a p-value adjusted with Bonferroni of 0.05. Genera are represented by nodes, and their size and color change according to their abundance and phylum, respectively. Green edges represent positive correlations, while red edges represent positive correlations, and the thicker the edge is, the stronger the Spearman correlation is.
